# Supplementary figures and images for: Transient CAR T cells with specificity to oncofetal glycosaminoglycans in solid tumors
Source: EMBO Mol Med. 2024 Oct 15;16(11):8. doi: 10.1038/s44321-024-00153-8 (PMC11554890; doi:10.1038/s44321-024-00153-8)

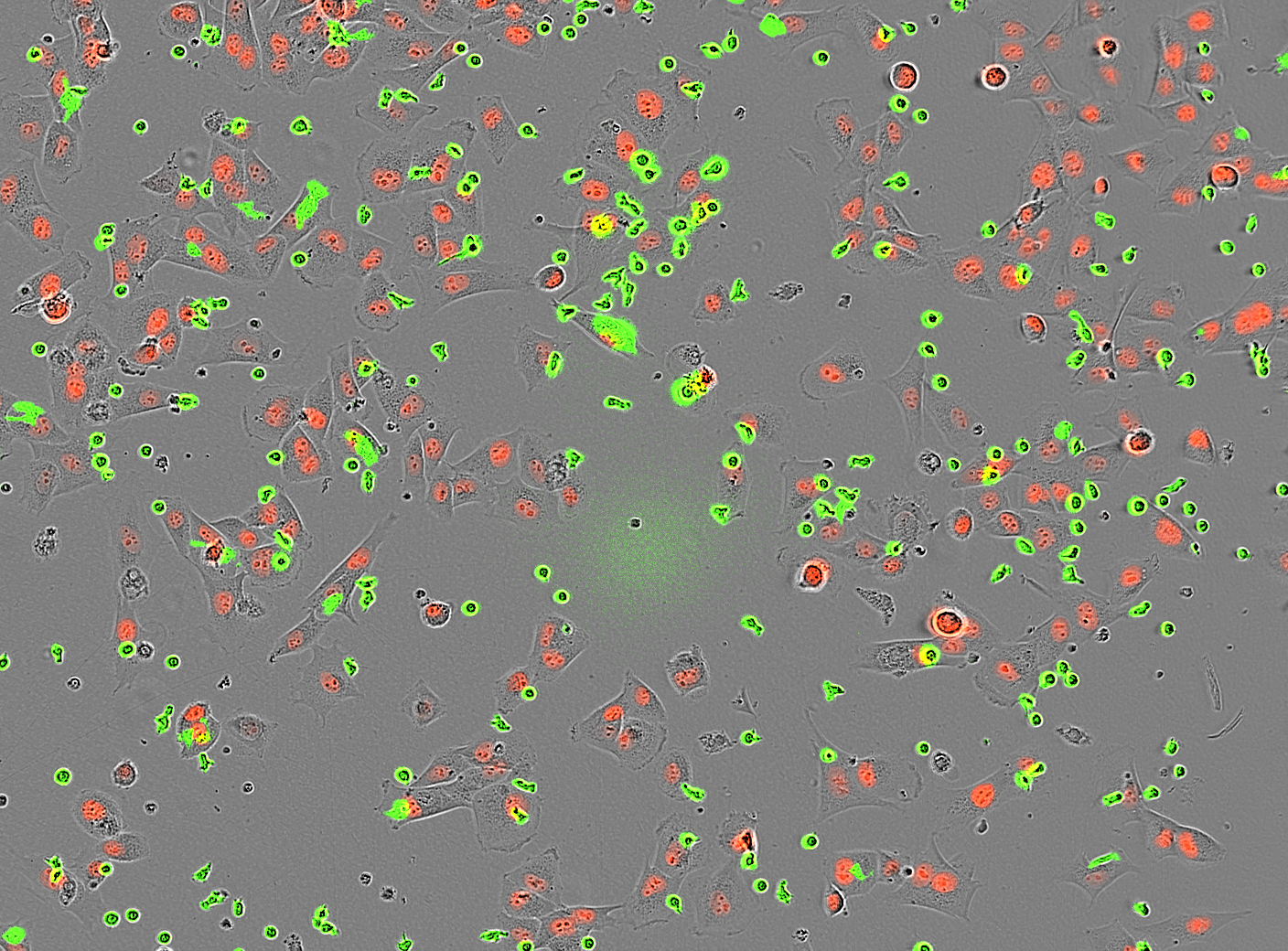

Supplement: Supplementary file 4 — Source data Fig. 3 [file 44321_2024_153_MOESM4_ESM.zip › Figure_3_-source_data/Figure 3 -source data/3A source data/MG63/ArmedCAR-MG63_day 0- no scale bar.tif]

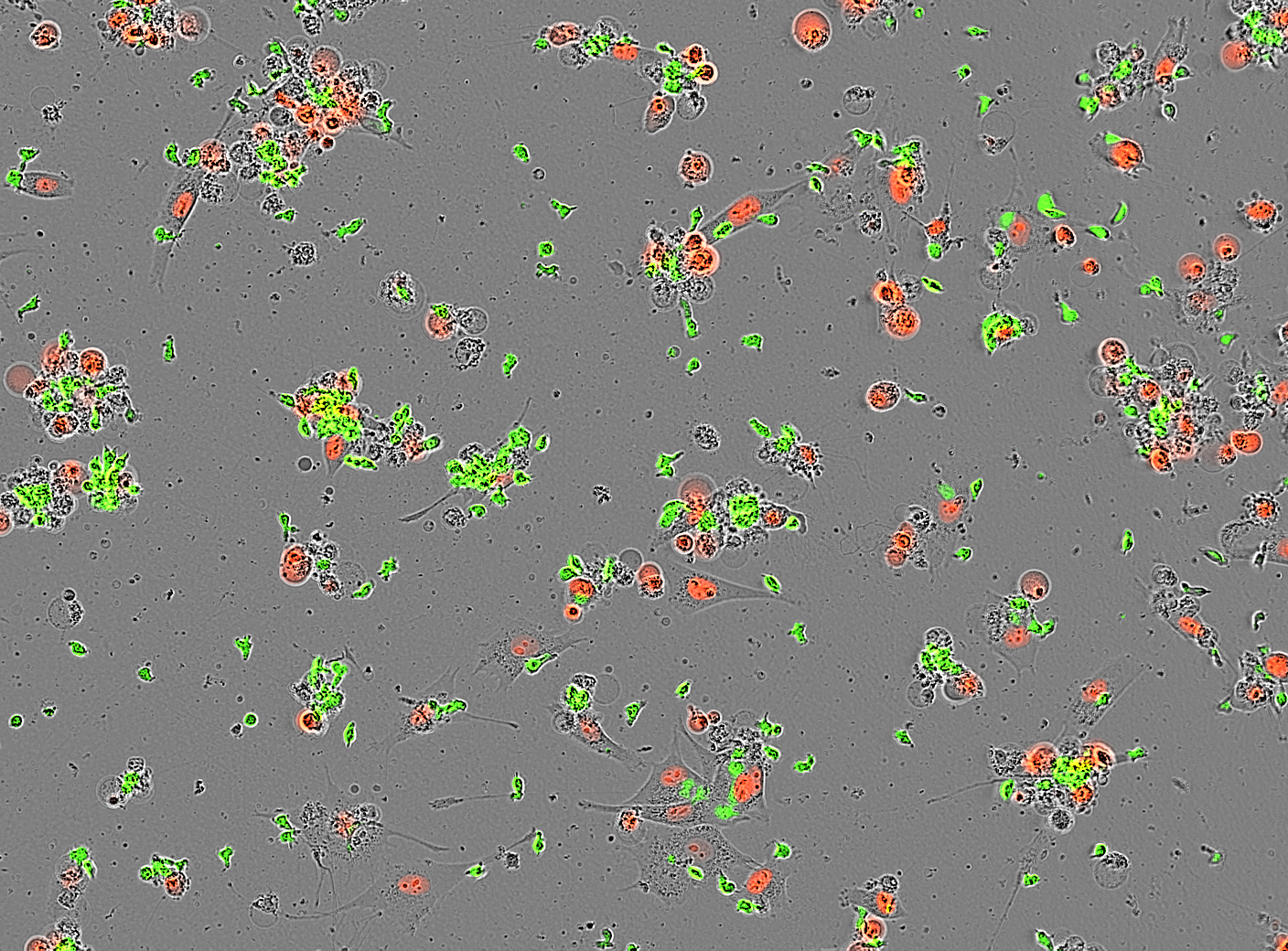

Supplement: Supplementary file 4 — Source data Fig. 3 [file 44321_2024_153_MOESM4_ESM.zip › Figure_3_-source_data/Figure 3 -source data/3A source data/MG63/ArmedCAR-MG63_day 1- no scale bar.tif]

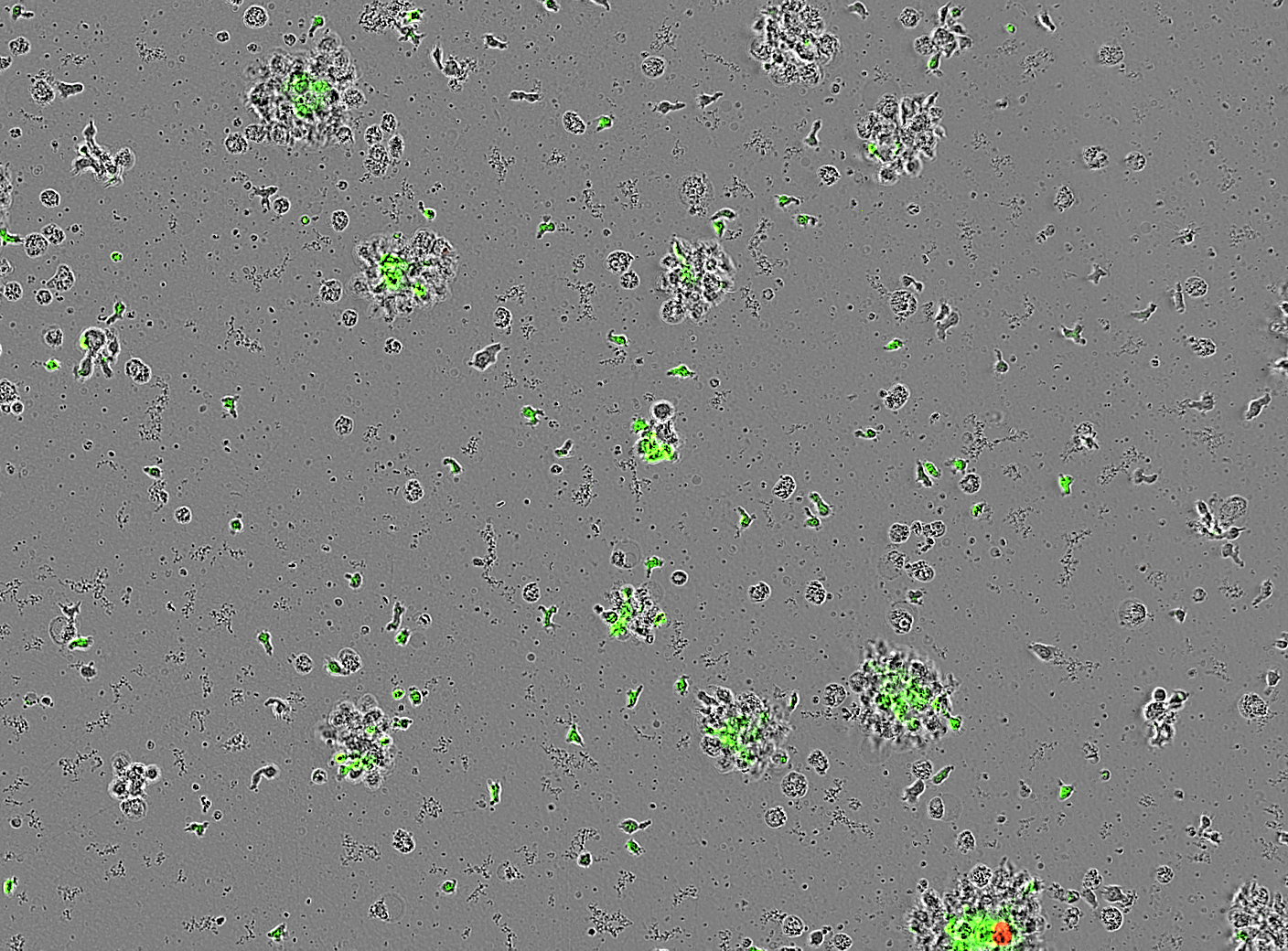

Supplement: Supplementary file 4 — Source data Fig. 3 [file 44321_2024_153_MOESM4_ESM.zip › Figure_3_-source_data/Figure 3 -source data/3A source data/MG63/ArmedCAR-MG63_day 2- no scale bar.tif]

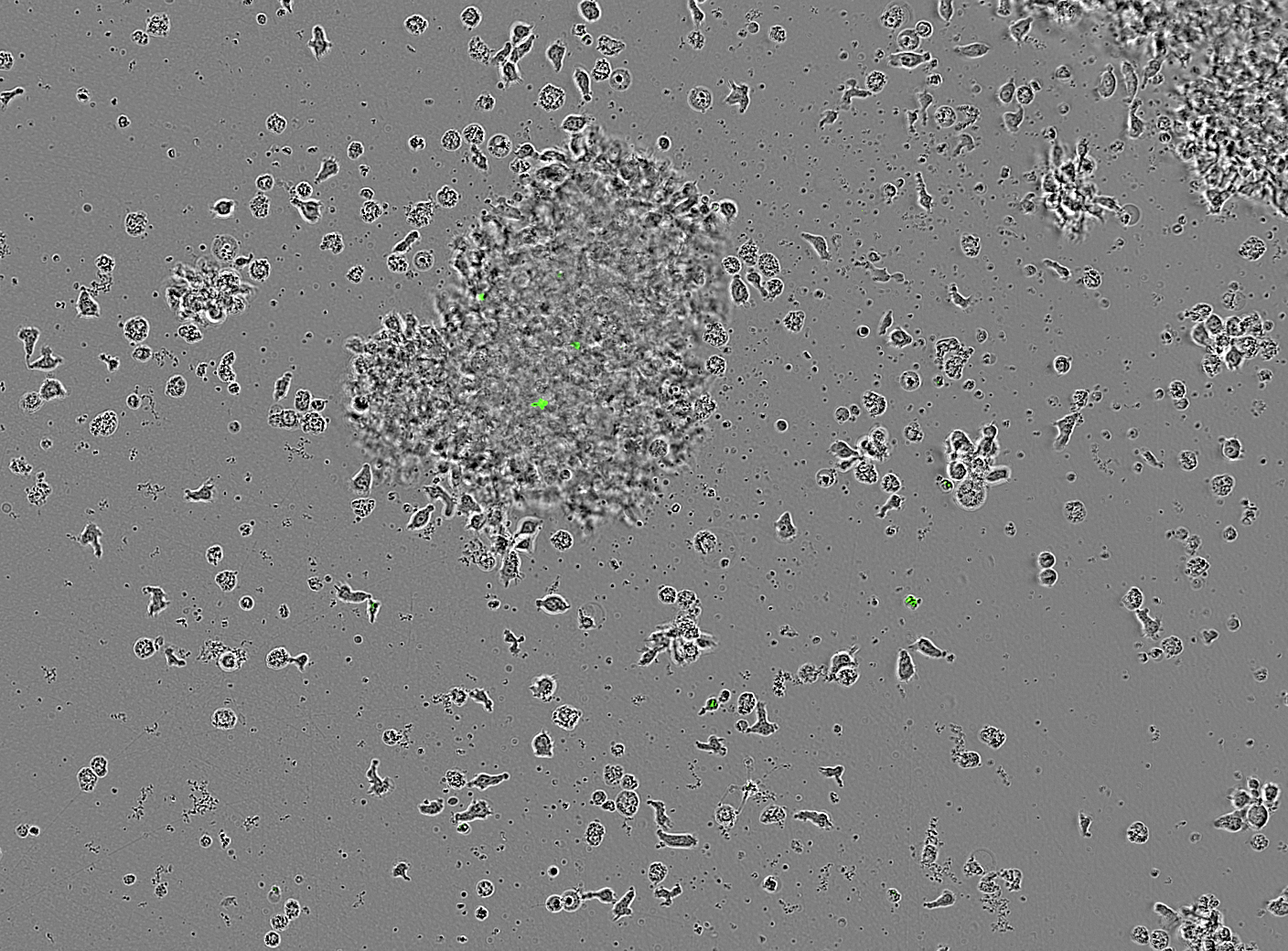

Supplement: Supplementary file 4 — Source data Fig. 3 [file 44321_2024_153_MOESM4_ESM.zip › Figure_3_-source_data/Figure 3 -source data/3A source data/MG63/ArmedCAR-MG63_day 3- no scale bar.tif]

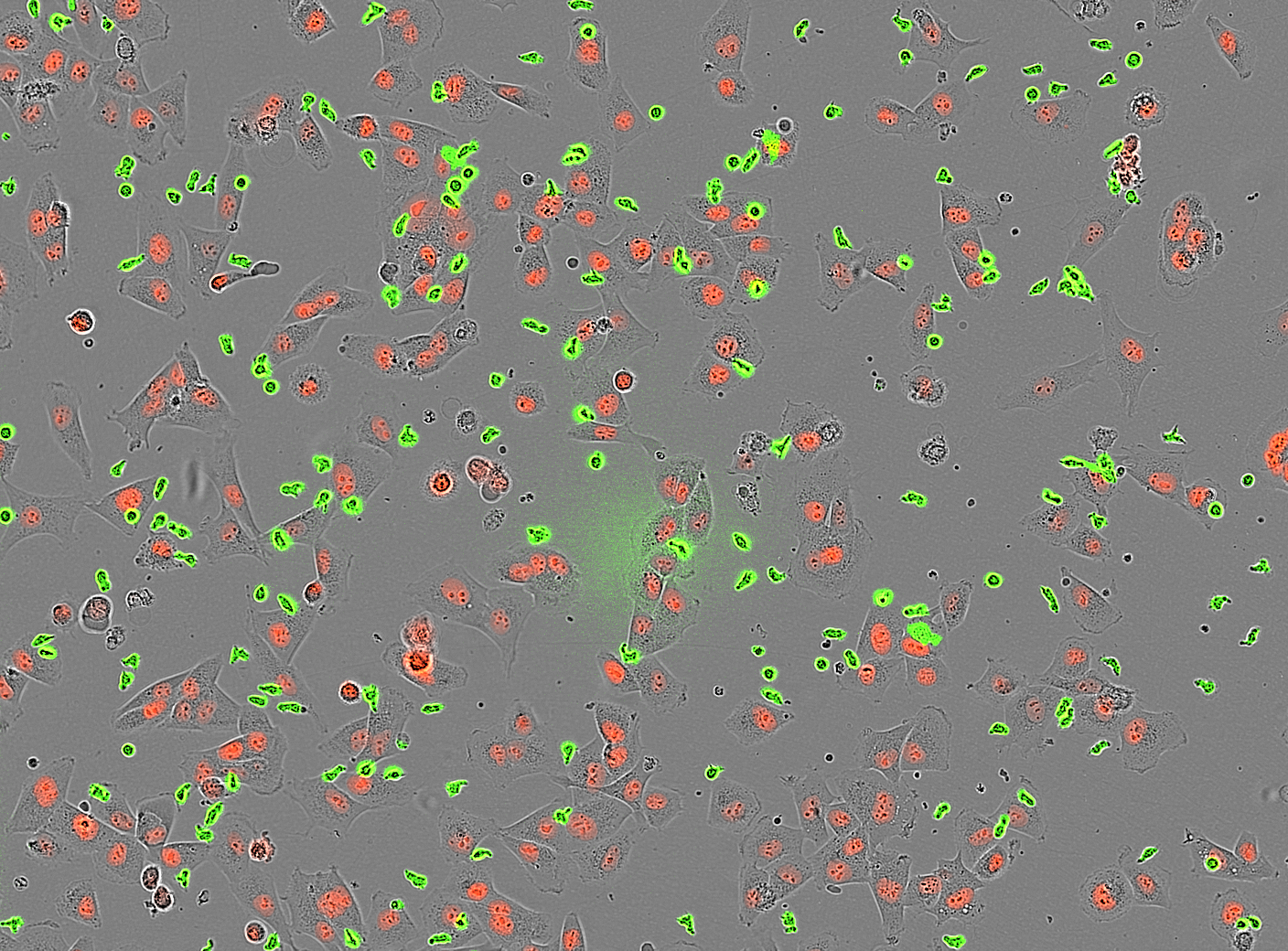

Supplement: Supplementary file 4 — Source data Fig. 3 [file 44321_2024_153_MOESM4_ESM.zip › Figure_3_-source_data/Figure 3 -source data/3A source data/MG63/UnarmedCAR-MG63_ day 0- no scale bar.tif]

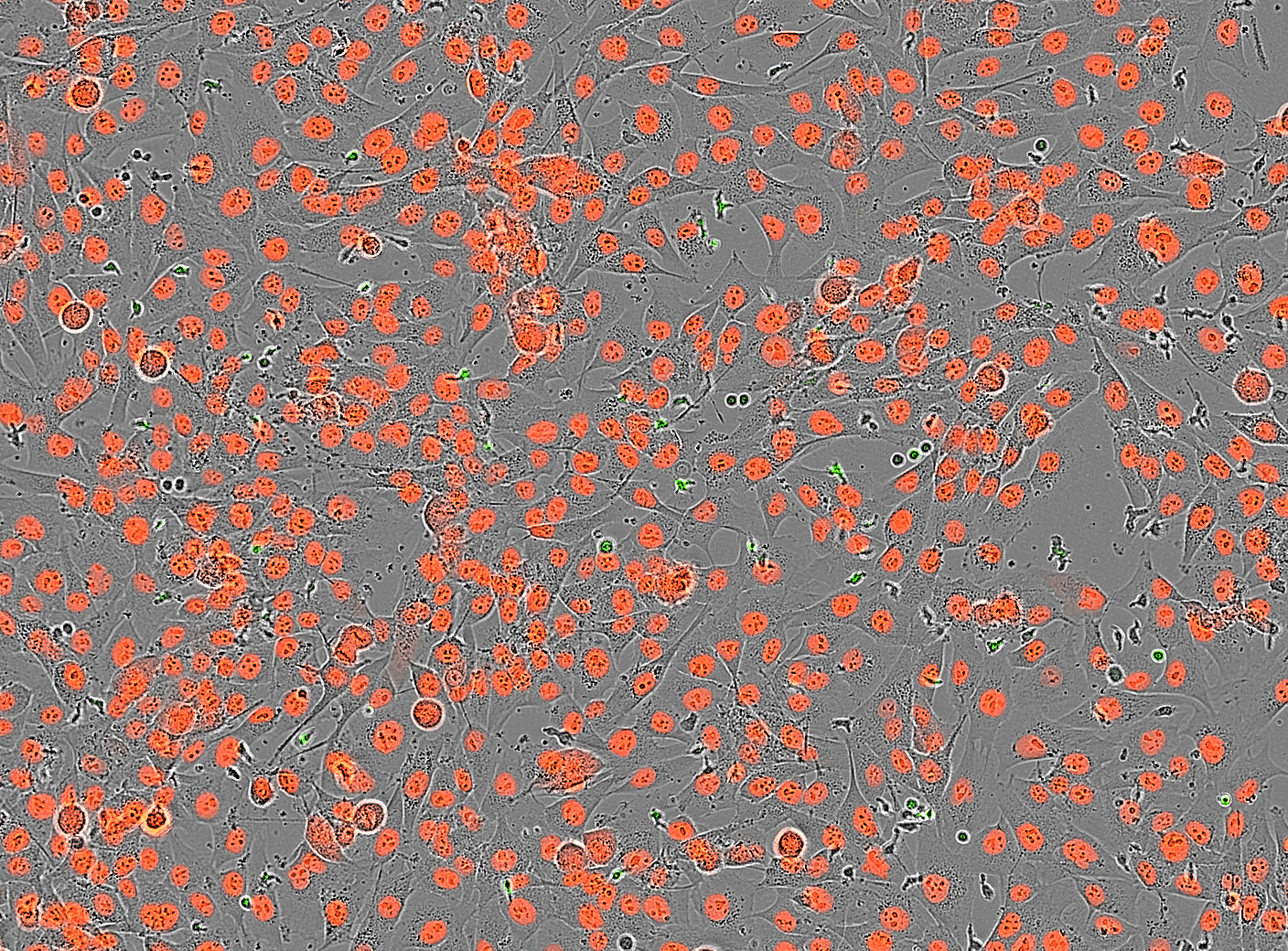

Supplement: Supplementary file 4 — Source data Fig. 3 [file 44321_2024_153_MOESM4_ESM.zip › Figure_3_-source_data/Figure 3 -source data/3A source data/MG63/UnarmedCAR-MG63_ day 2- no scale bar.tif]

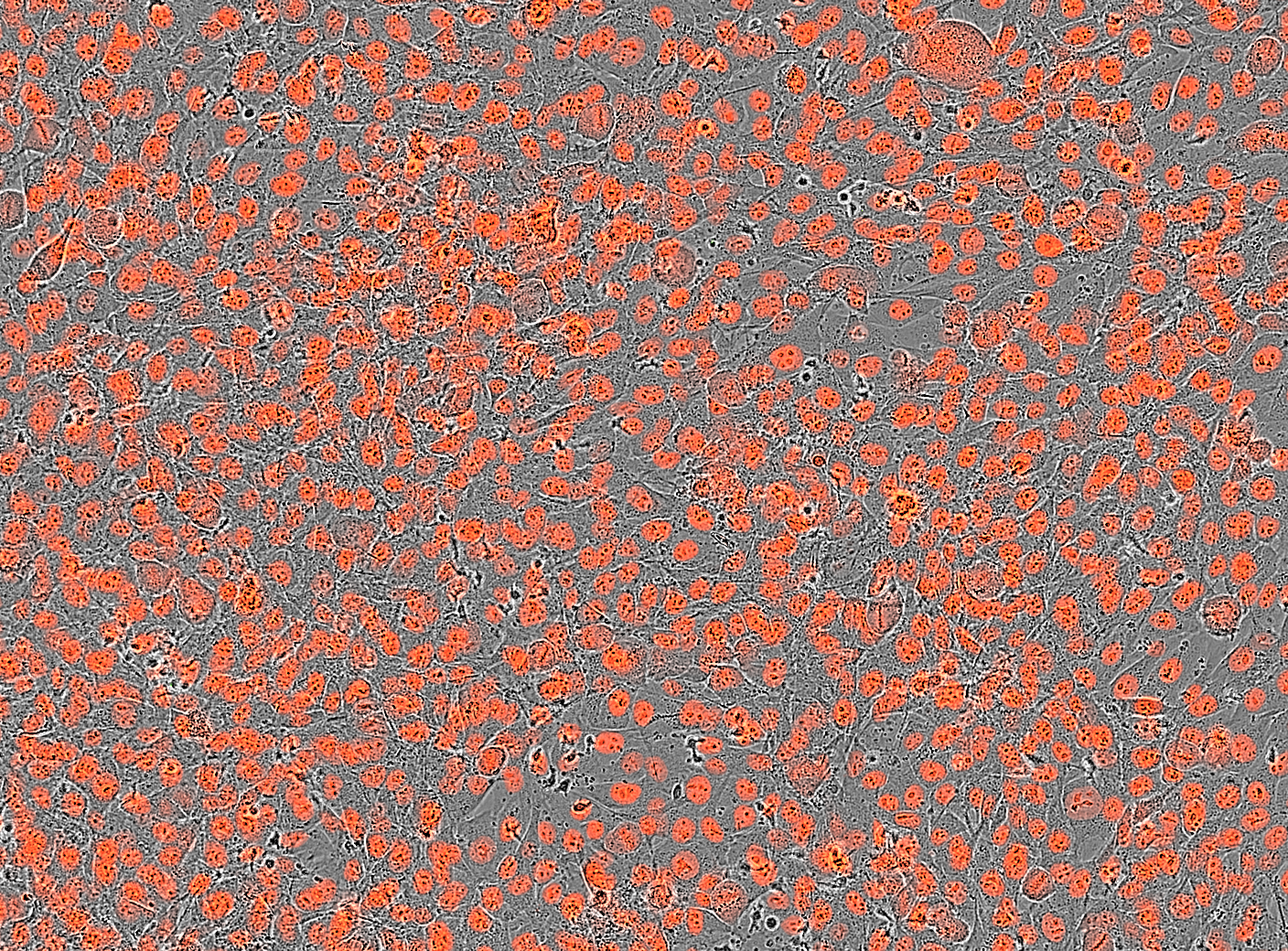

Supplement: Supplementary file 4 — Source data Fig. 3 [file 44321_2024_153_MOESM4_ESM.zip › Figure_3_-source_data/Figure 3 -source data/3A source data/MG63/UnarmedCAR-MG63_ day 3- no scale bar.tif]

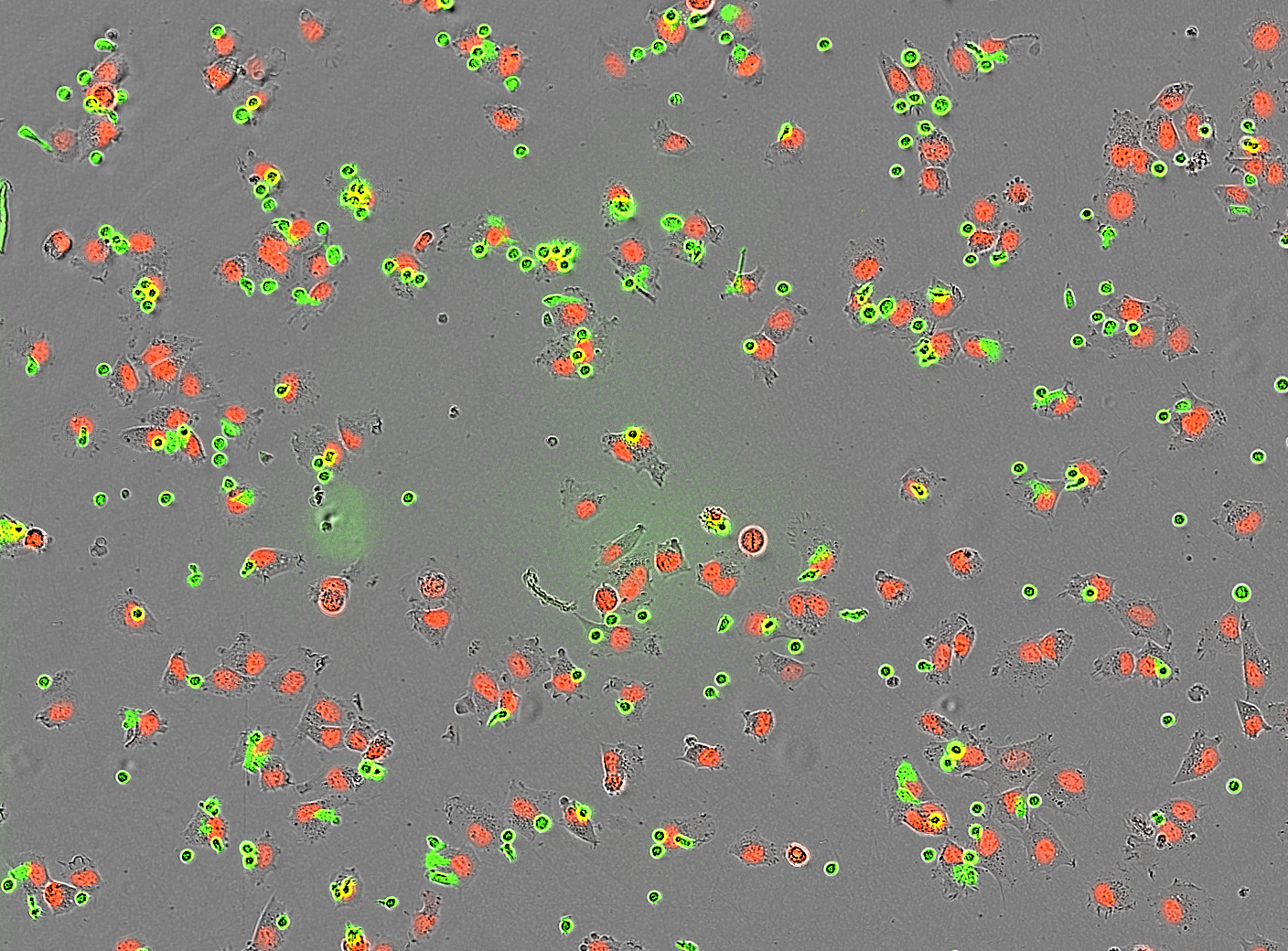

Supplement: Supplementary file 4 — Source data Fig. 3 [file 44321_2024_153_MOESM4_ESM.zip › Figure_3_-source_data/Figure 3 -source data/3A source data/UC3/ArmedCAR-UC3_day 0-no scale.tif]

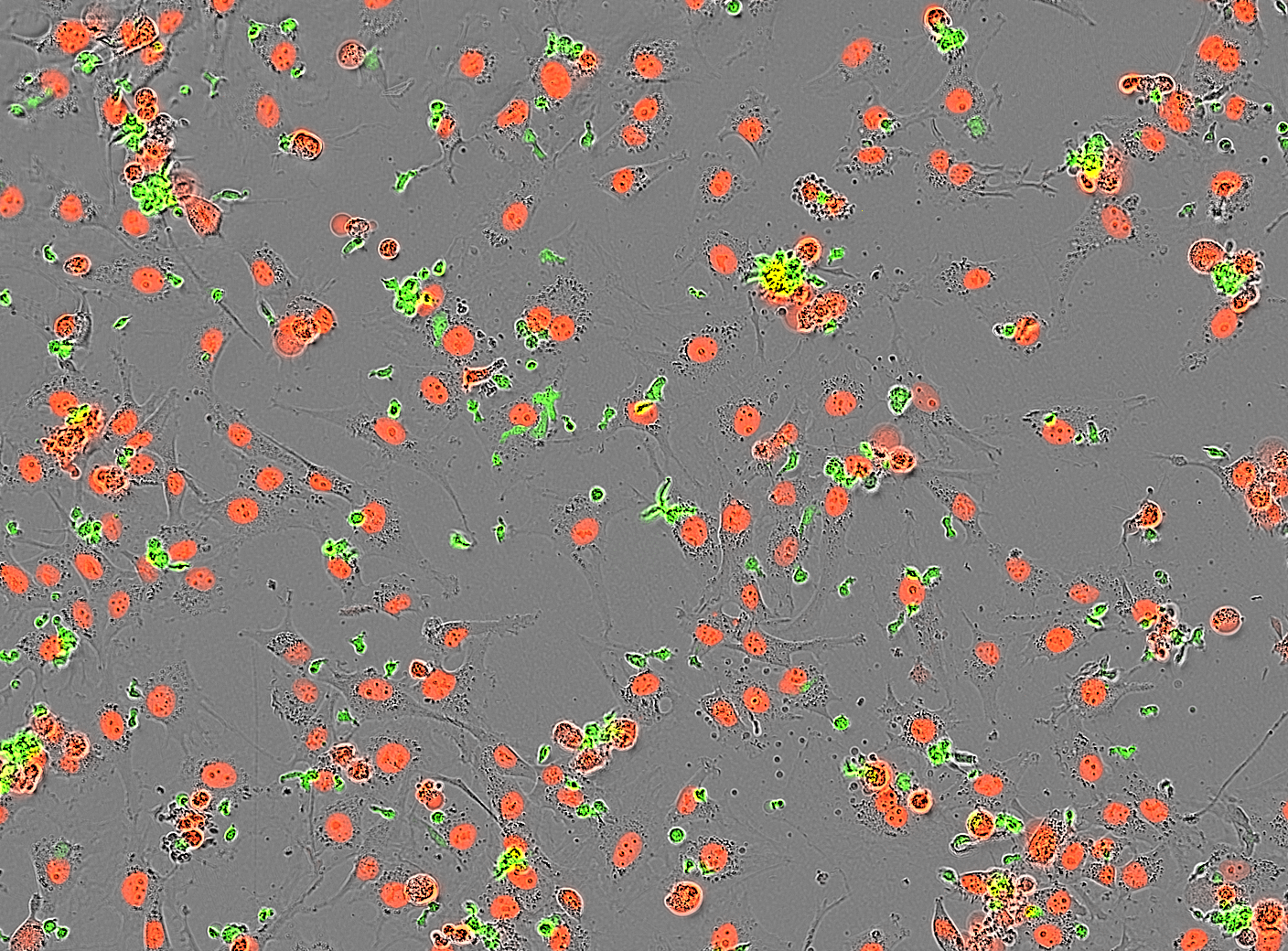

Supplement: Supplementary file 4 — Source data Fig. 3 [file 44321_2024_153_MOESM4_ESM.zip › Figure_3_-source_data/Figure 3 -source data/3A source data/UC3/ArmedCAR-UC3_day 1-no scale.tif]

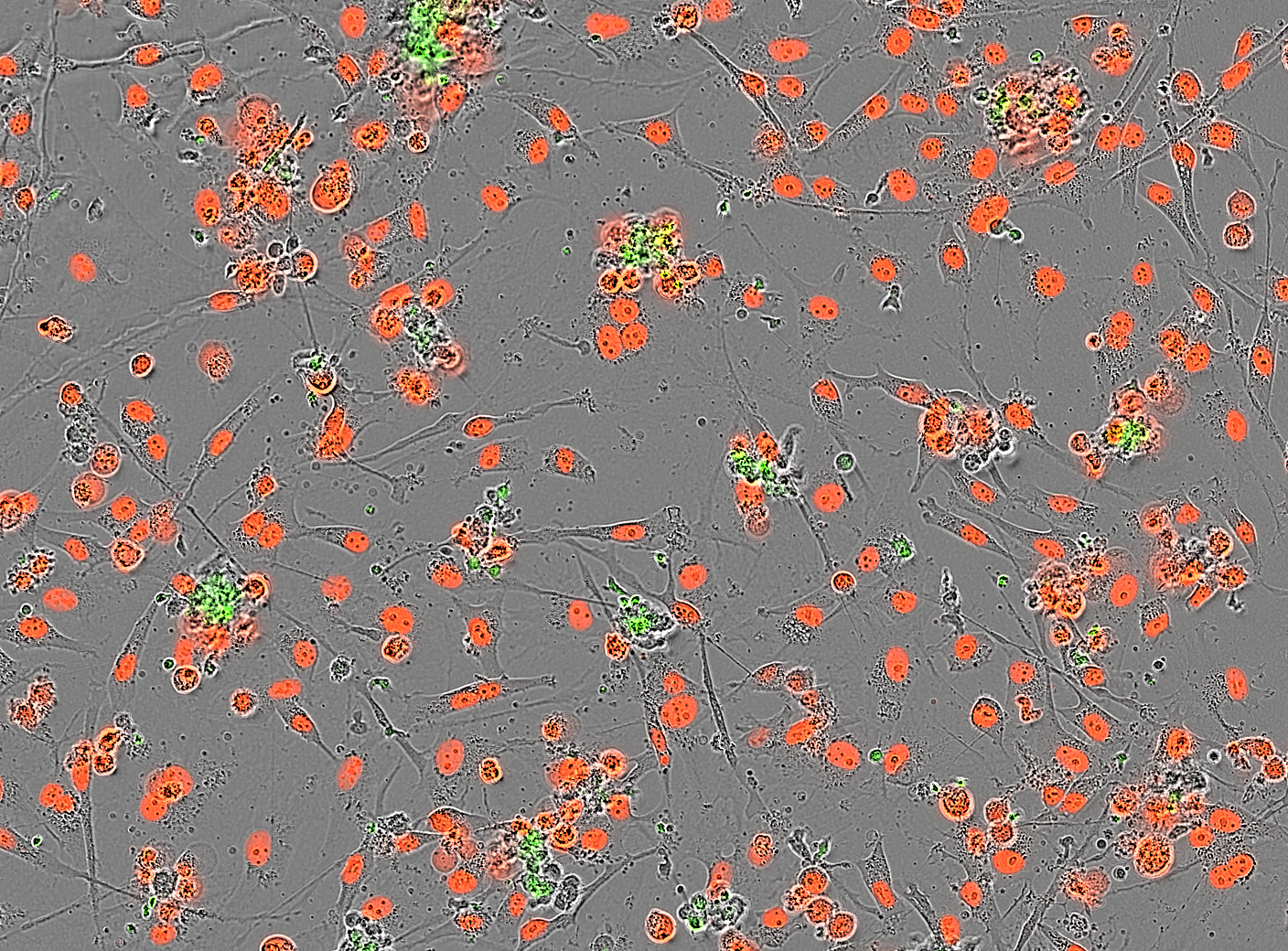

Supplement: Supplementary file 4 — Source data Fig. 3 [file 44321_2024_153_MOESM4_ESM.zip › Figure_3_-source_data/Figure 3 -source data/3A source data/UC3/ArmedCAR-UC3_day 2-no scale.tif]

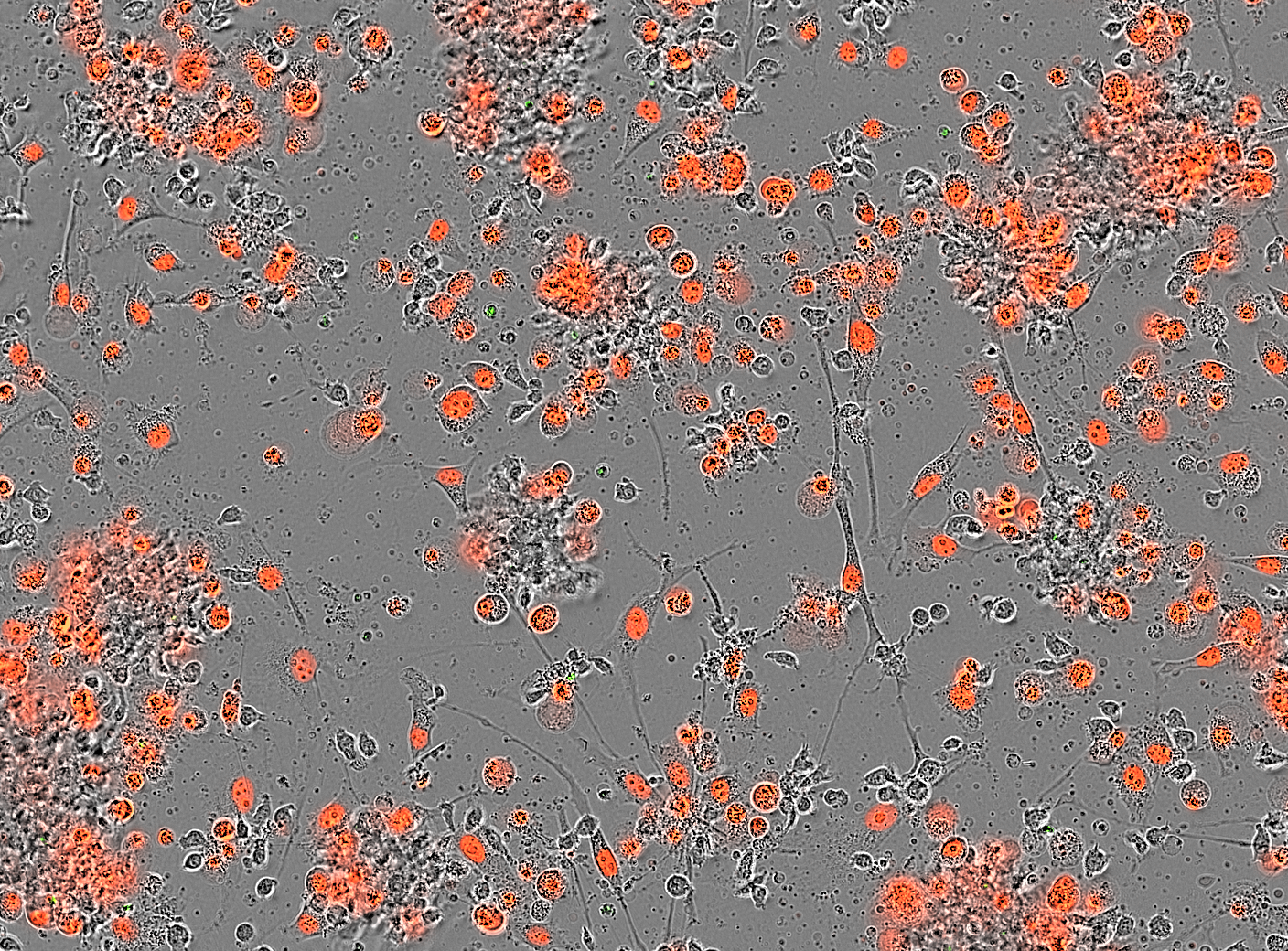

Supplement: Supplementary file 4 — Source data Fig. 3 [file 44321_2024_153_MOESM4_ESM.zip › Figure_3_-source_data/Figure 3 -source data/3A source data/UC3/ArmedCAR-UC3_day 3-no scale.tif]

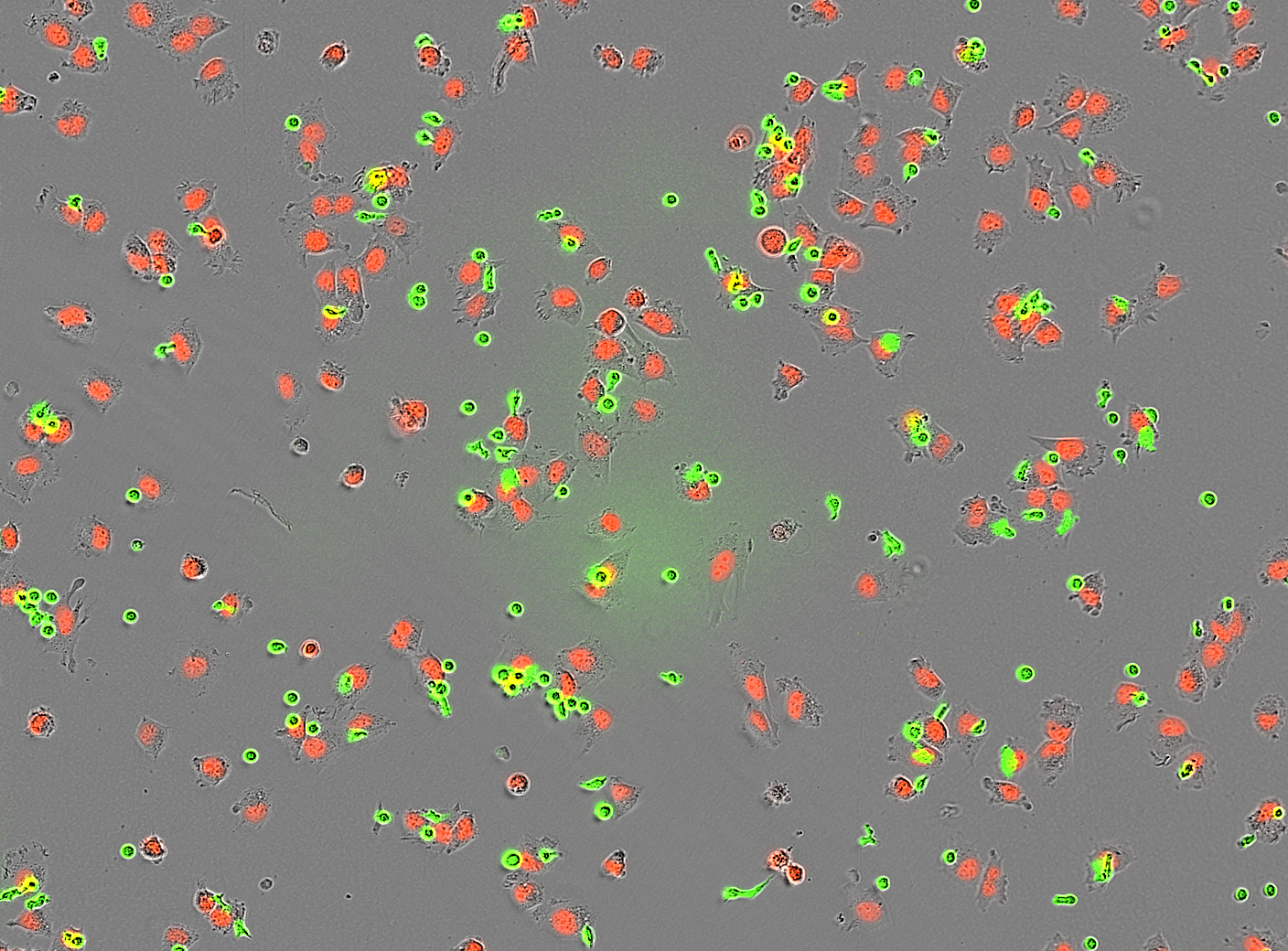

Supplement: Supplementary file 4 — Source data Fig. 3 [file 44321_2024_153_MOESM4_ESM.zip › Figure_3_-source_data/Figure 3 -source data/3A source data/UC3/UnarmedCAR-UC3_day 0- no scale bar.tif]

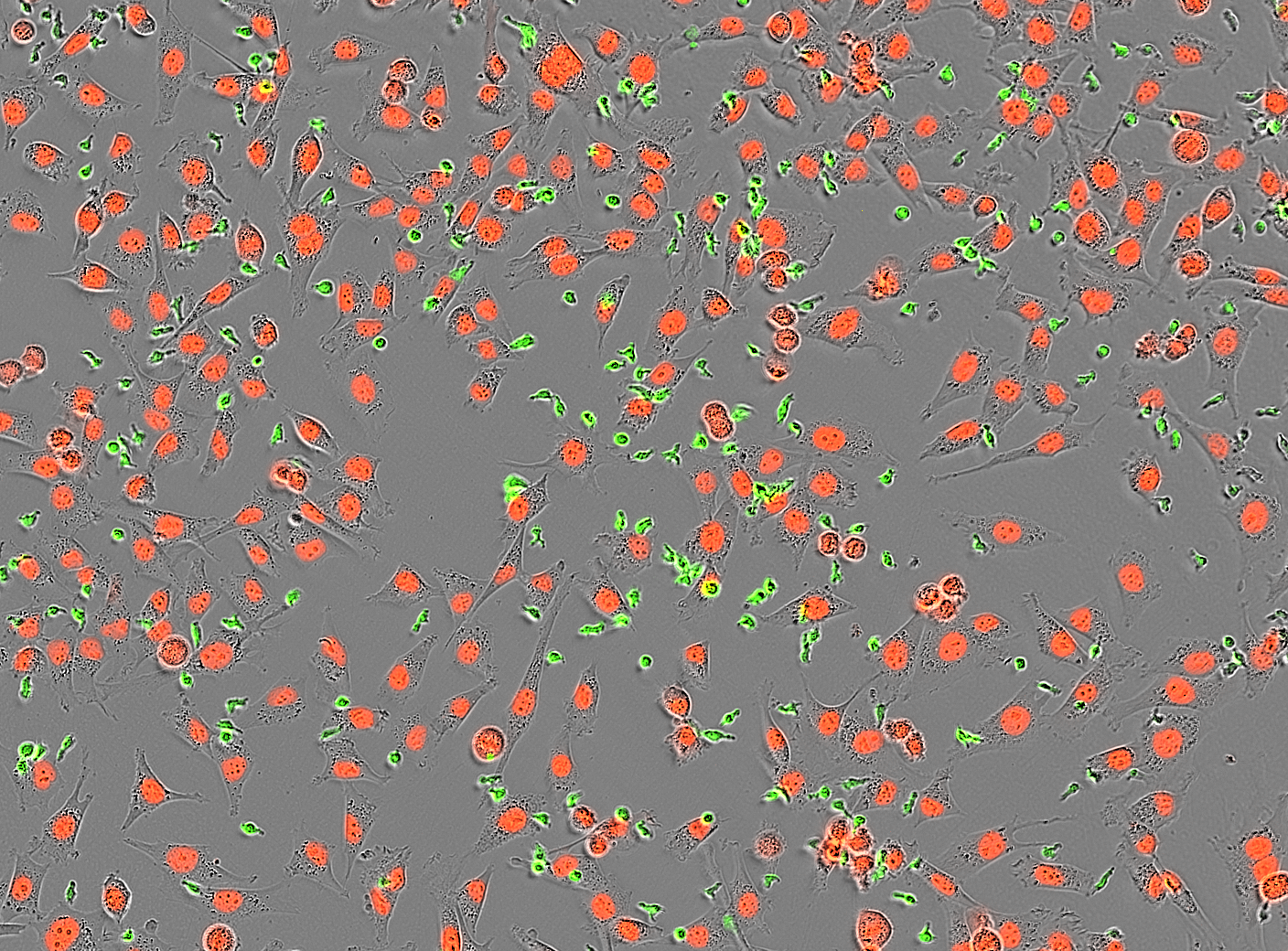

Supplement: Supplementary file 4 — Source data Fig. 3 [file 44321_2024_153_MOESM4_ESM.zip › Figure_3_-source_data/Figure 3 -source data/3A source data/UC3/UnarmedCAR-UC3_day 1- no scale bar.tif]

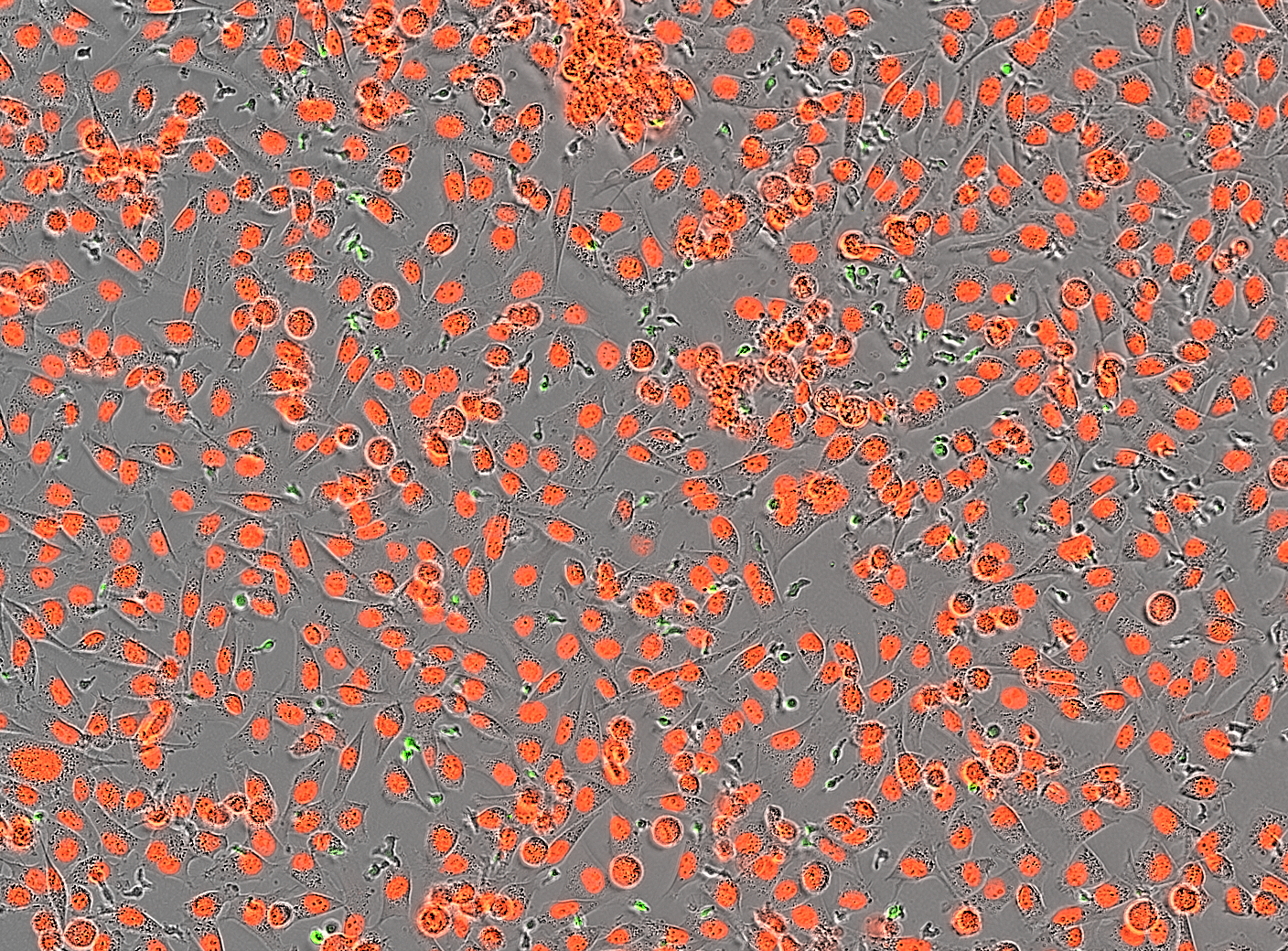

Supplement: Supplementary file 4 — Source data Fig. 3 [file 44321_2024_153_MOESM4_ESM.zip › Figure_3_-source_data/Figure 3 -source data/3A source data/UC3/UnarmedCAR-UC3_day 2- no scale bar.tif]

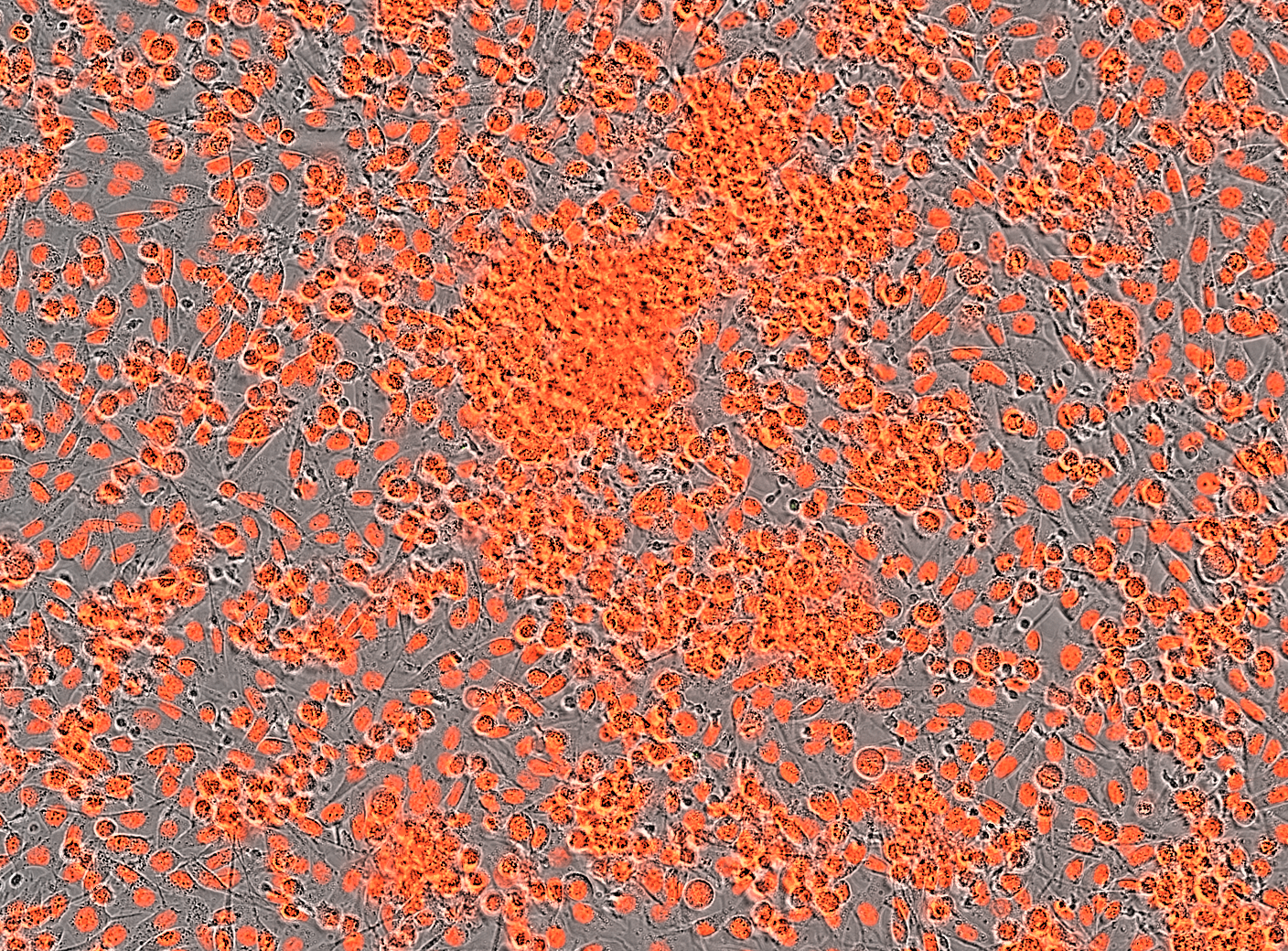

Supplement: Supplementary file 4 — Source data Fig. 3 [file 44321_2024_153_MOESM4_ESM.zip › Figure_3_-source_data/Figure 3 -source data/3A source data/UC3/UnarmedCAR-UC3_day 3- no scale bar.tif]
