# Supplementary material for: Transient CAR T cells with specificity to oncofetal glycosaminoglycans in solid tumors
Source: EMBO Mol Med. 2024 Oct 15;16(11):8. doi: 10.1038/s44321-024-00153-8 (PMC11554890; doi:10.1038/s44321-024-00153-8)
Supplement: Supplementary file 7 — Expanded View Figures [file 44321_2024_153_MOESM7_ESM.pdf]

Expanded View Figures

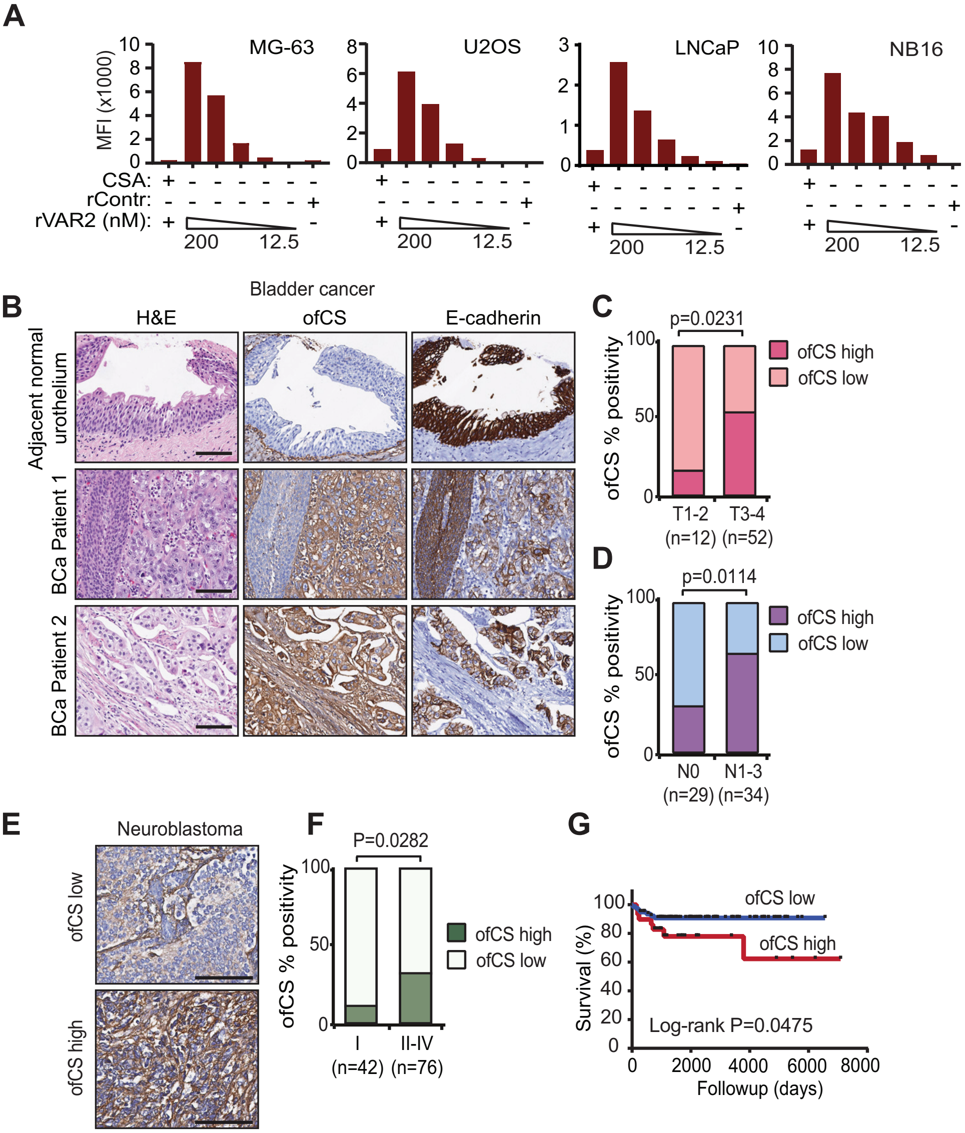

Figure EV1. Oncofetal CS expression in solid tumor cell lines and bladder cancer tissue.

(**A**) MG-63, U2OS, LNCaP, and NB16 tumor cell lines were incubated with indicated concentrations of rContr protein or VAR2 (12–200 nM) +/− purified CSA and analyzed by flow cytometry using anti-V5-FITC. (**B**) Representative H&E and IHC images of normal adjacent urothelium and bladder cancer tissues from two patients. Matched staining images of E-cadherin, as an epithelial marker, in parallel with oncofetal CS (ofCS) detection by VAR2 and anti-V5. (**C**) Bar plot of bladder cancer patient tumors ($n = 64$) indicating ofCS expression in relation to T stage. (**D**) Bar plot of bladder cancer patient tumors ($n = 63$) indicating ofCS expression in relation to N stage. (**E**) Representative IHC images of neuroblastoma tumors selected for high and low oncofetal CS expression. (**F**) Percent ofCS-positive neuroblastoma tumors related to tumor stage. (**G**) Kaplan–Meier plot indicating overall survival of neuroblastoma patients related to oncofetal CS expression. The scale bar represents 100 μm. MIBC: muscle-invasive bladder cancer; oncofetal CS: oncofetal chondroitin sulfate. For statistical analysis in the above panels (C: T stage; D: N stage and F: International Neuroblastoma Staging System (INSS)), two-tailed Fisher's exact test was used. MFI Mean Fluorescence Intensity, CSA chondroitin sulfate A, OfCS Oncofetal Chondroitin Sulfate.

© The Author(s)

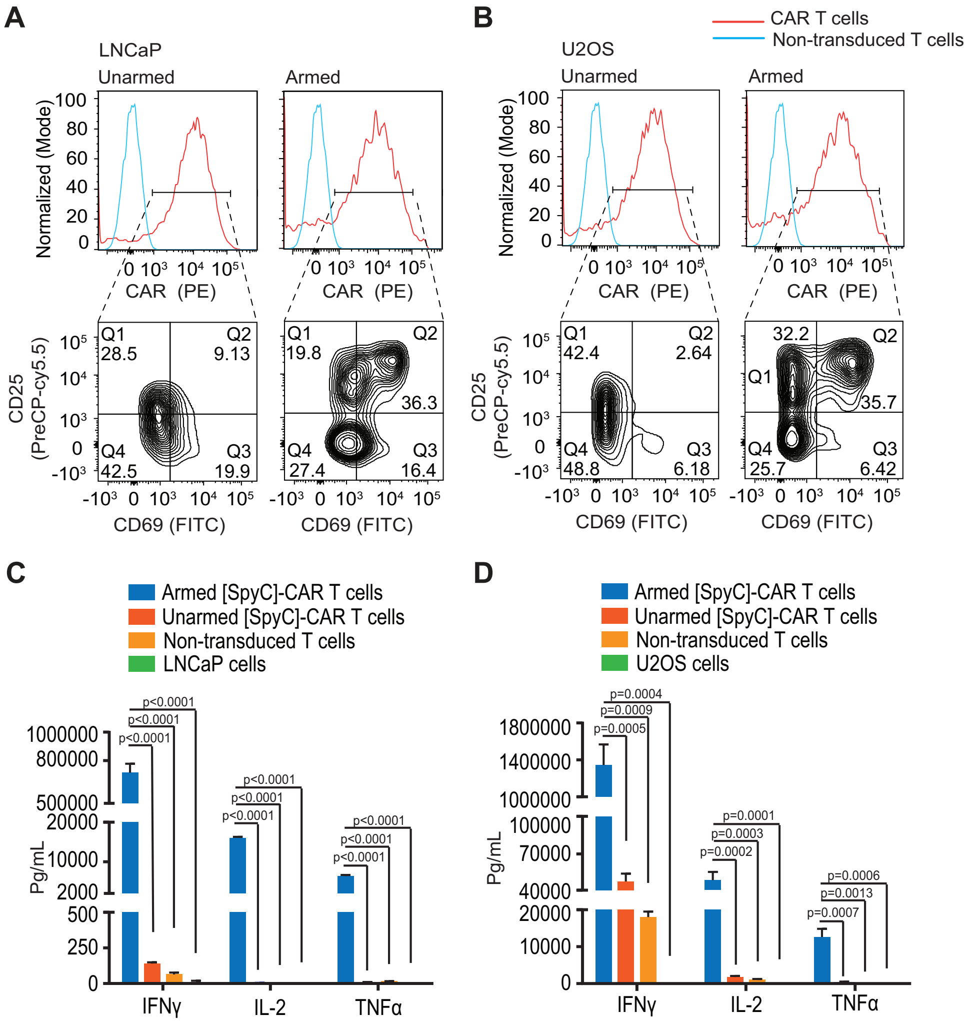

Figure EV2. Activation of VAR2-[SpyT][SpyC]-CAR T cells upon tumor cell engagement.

(**A**, **B**) Armed and unarmed [SpyC]-CAR T cells were incubated with (**A**) LNCaP and (**B**) U2OS cells at a 1:1 E:T ratio for 24 h before analyzed for Flag, CD69, and CD25 expression by flow cytometry. The results are representative of 3 independent experiments. (**C, D**) Armed and unarmed [SpyC]-CAR T cells were incubated in triplicate, with (**C**) LNCaP and (**D**) U2OS cells at a 10:1 E:T ratio in 100 µl media, for 48 h and analyzed for the concentration of indicated cytokines in the culture supernatant. Results are presented as mean ± SEM of three different wells. The statistical significance was determined using one-way ANOVA followed by Dunnett's multiple comparison's test; *P* values for each comparison are indicated in the figure. E:T effector-to-target cell ratio.

 © The Author(s)

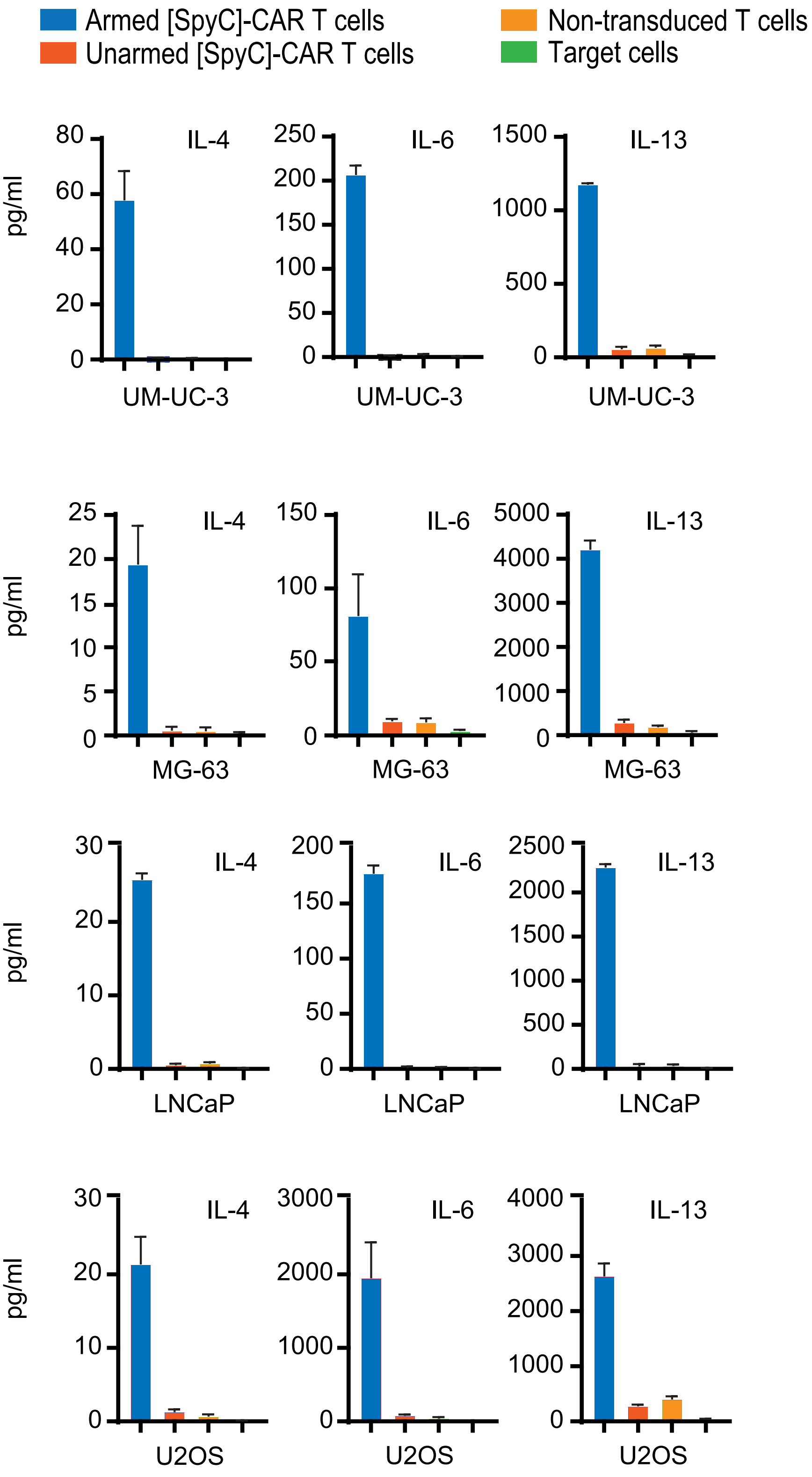

Figure EV3. Cytokine responses in co-cultures of effector and target cells.

The concentrations of the indicated cytokines in the culture supernatants were assessed after 48 h of co-culturing between effector T cells formulations and different target cells (i.e., UM-UC3, MG-63, LNCaP, and U2OS), at a 10:1 E:T ratio. Data was analyzed with the Discovery Workbench software. Error bars represent mean ± SEM of three different wells. E:T effector-to-target cell ratio.

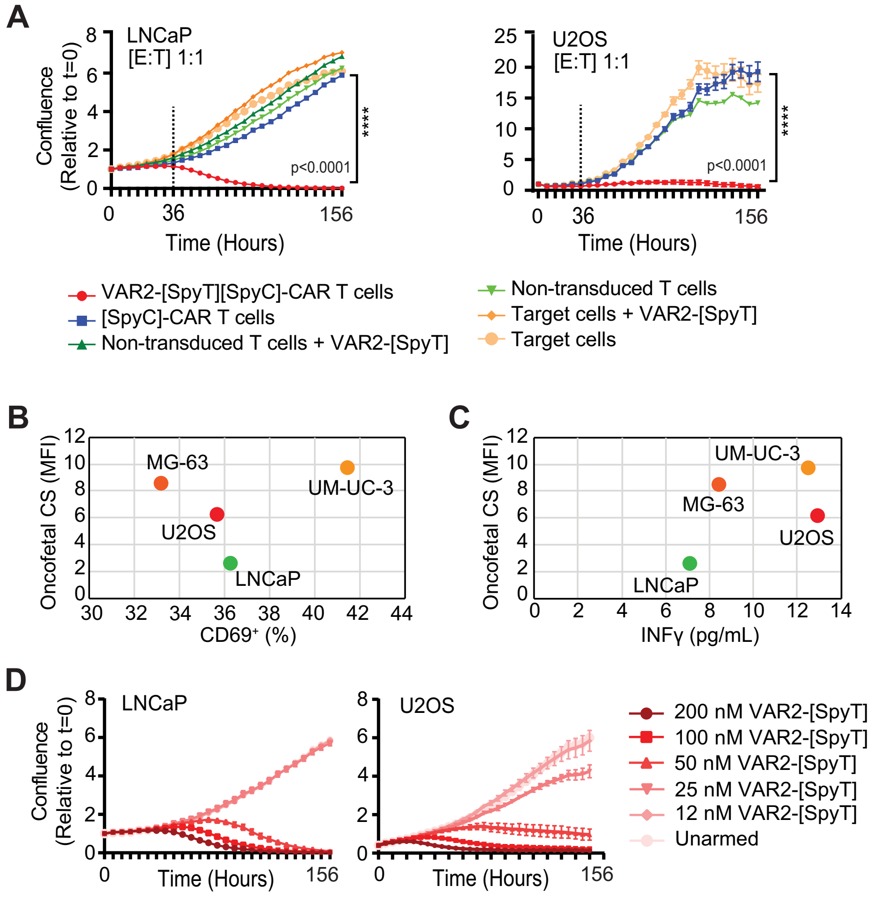

Figure EV4. Activity of VAR2-[SpyT][SpyC]-CAR T cells after target cell engagement.

(**A**) LNCaP and U2OS target cells (red) were co-cultured in triplicate with indicated formulations of T cells and monitored for one week. Dashed lines indicate time to VAR2-[SpyT][SpyC]-CAR T-cell cytotoxicity. Error bars represent mean ± SEM of triplicate wells. Data from a representative donor of three individual donors is shown. Statistical analyses were performed at the final timepoint using one-way ANOVA with Dunnett's multiple comparisons test. The cytotoxicity of VAR2-[SpyT][SpyC]-CAR T cells compared to all other groups in both target cell lines showed a *P* value of $P < 0.0001$. (**B**) Percent CD69-positive VAR2-[SpyT][SpyC]-CAR T cells plotted against oncofetal CS expression in indicated target cells. (**C**) IFNγ production (pg/ml) in co-cultures of VAR2-[SpyT][SpyC]-CAR T cells and indicated target cells plotted against oncofetal CS expression of the target cells. All data was analyzed by GraphPad Prism Software. (**D**) LNCaP and U2OS target cells (red) were co-cultured with [SpyC]-CAR T cells at a 1:1 E:T ratio with indicated concentrations of VAR2-[SpyT] protein and analyzed for viability using confluence as the readout. Error bars represent mean ± SEM of triplicate wells. E:T effector-to-target cell ratio, MFI Mean Fluorescence Intensity.

© The Author(s)

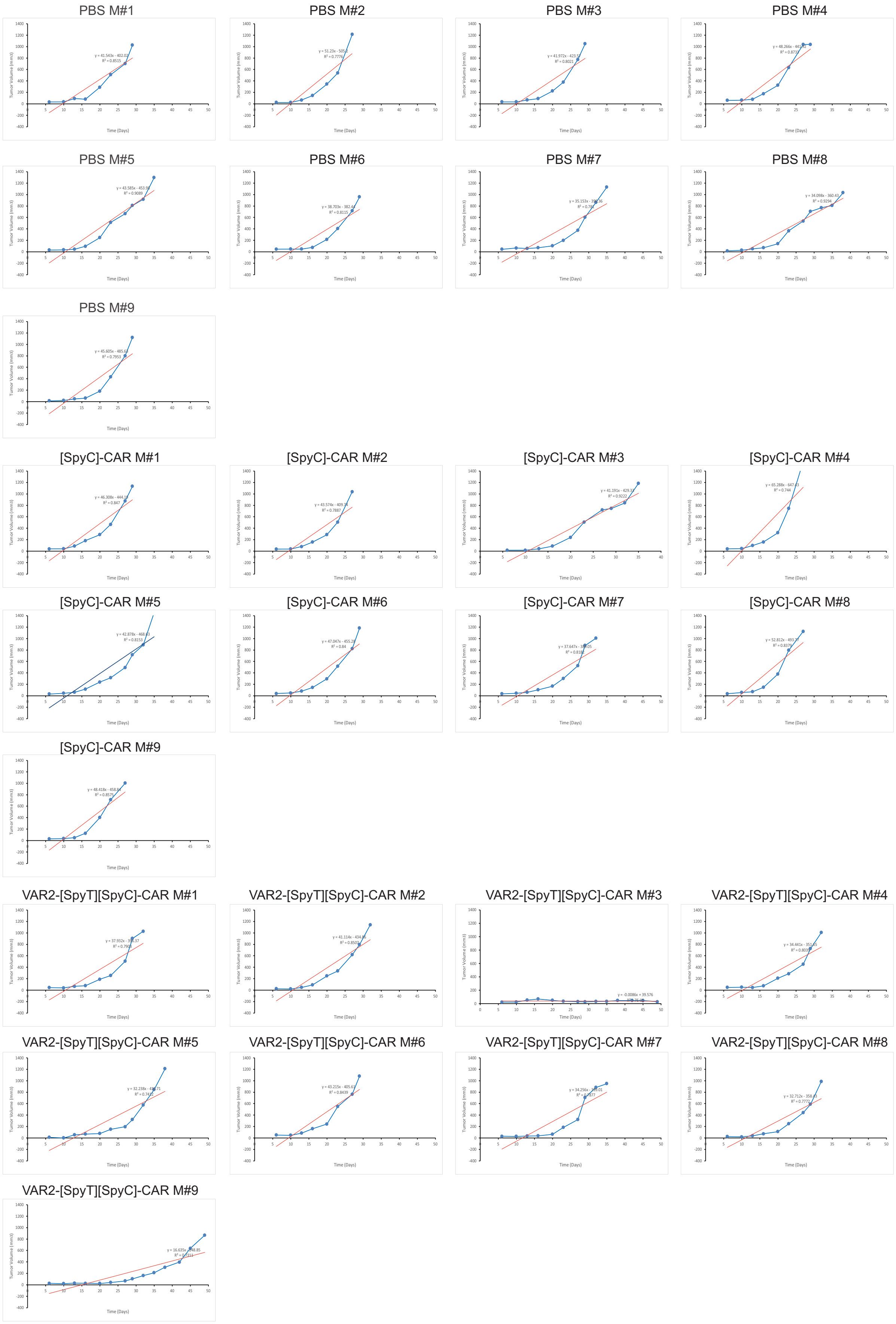
PBS M#1
PBS M#2
PBS M#3
PBS M#4
PBS M#5
PBS M#6
PBS M#7
PBS M#8
PBS M#9
[SpyC]-CAR M#1
[SpyC]-CAR M#2
[SpyC]-CAR M#3
[SpyC]-CAR M#4
[SpyC]-CAR M#5
[SpyC]-CAR M#6
[SpyC]-CAR M#7
[SpyC]-CAR M#8
[SpyC]-CAR M#9
VAR2-[SpyT][SpyC]-CAR M#1
VAR2-[SpyT][SpyC]-CAR M#2
VAR2-[SpyT][SpyC]-CAR M#3
VAR2-[SpyT][SpyC]-CAR M#4
VAR2-[SpyT][SpyC]-CAR M#5
VAR2-[SpyT][SpyC]-CAR M#6
VAR2-[SpyT][SpyC]-CAR M#7
VAR2-[SpyT][SpyC]-CAR M#8
VAR2-[SpyT][SpyC]-CAR M#9
Tumor Volume (mm3)
Time (Days)

Figure EV5. Linear regression analysis of tumor growth.

Individual tumor growth curve (blue line) and the slope of the curve (red line) is shown for each mouse treated with PBS, [SpyC]-CAR T cells or VAR2-[SpyT][SpyC]-CAR T cells.

 © The Author(s)
